# Hearing Outcomes from Gamma Knife Treatment for Intracanalicular Vestibular Schwannomas with Good Initial Hearing

**DOI:** 10.3390/jcm13061685

**Published:** 2024-03-14

**Authors:** Philippine Toulemonde, Nicolas Reyns, Michael Risoud, Pierre-Emmanuel Lemesre, Frédéric Gabanou, Marc Baroncini, Jean-Paul Lejeune, Rabih Aboukais, Christophe Vincent

**Affiliations:** 1 Otology and Neurotology Department, CHU de Lille, 59037 Lille, Francepierreemmanuel.lemesre@chru-lille.fr (P.-E.L.); frederic.gabanou@ghsc.fr (F.G.);; 2 Neurosurgery Department, CHU de Lille, 59037 Lille, Francemarc.baroncini@chru-lille.fr (M.B.);

**Keywords:** vestibular schwannoma, gamma knife, stereotactic radiotherapy, hearing

## Abstract

**Background:** The objective of this study was to describe the long-term hearing outcomes of gamma knife treatment for unilateral progressing vestibular schwannomas (VS) presenting with good initial hearing using audiologic data. **Methods:** A retrospective review was performed between 2010 and 2020 to select patients with progressing unilateral VS and good hearing (AAO-HNS class A) treated with stereotactic gamma knife surgery (GKS). Their audiograms were analyzed along with treatment metrics and patient data. **Results**: Hearing outcomes with a median follow-up of 5 years post-treatment showed statistically significant loss of serviceable hearing: 34.1% of patients maintained good hearing (AAO-HNS class A), and 56.1% maintained serviceable hearing (AAO-HNS class A and B). Non-hearing outcomes are favorable with excellent tumor control and low facial nerve morbidity. **Conclusions:** Hearing declines over time in intracanalicular VS treated with GKS, with a significant loss of serviceable hearing after 5 years. The mean cochlear dose and the presence of cochlear aperture obliteration by the tumor are the main statistically significant factors involved in the hearing outcomes.

## 1. Introduction

Vestibular schwannomas (VS) are benign tumors growing from the Schwann cells of the vestibular nerve, accounting for 6 to 8% of all intracranial tumors [1]. Incidence rates were reported to have increased over the years due to the enhancement of clinical awareness and imaging standards, and more intracanalicular VS have been detected.

These patients usually present with unilateral hearing loss or other otological symptoms (unilateral tinnitus, vertigo) while neurological symptoms tend to manifest in cases with larger tumors [1].

However, no present consensus defines the management of these small VS (also known as Koos stage 1 tumors) among the “wait and scan” approach, surgical resection mainly via the middle fossa approach (MFA), and GKS [2]. GKS has emerged as a validated treatment option in the management of small VS because of its high tumor control rate and low morbidity. In most series, facial nerve deficit is reported in the range of 0% to 1% [2]. Comparing the tumor volume after GKS and the natural history of these patients remains a debated topic, especially if there is no evidence of tumor progression before GKS. Moreover, the definition of tumor progression varies across studies: a maximum tumor diameter increase of >20%, a tumor volume increase of 10%, ≥20%, or ≥25%, a maximum growth of the diameter of >2 mm; 1 mm of tumor growth in two directions or 2 mm of tumor growth in one direction; a minimum diameter increase of 2 mm in any direction; and a tumor more than 5 mm along any axis or 25% larger compared to any baseline dimension [2].

Despite the high tumor control rate and low morbidity, predicting hearing preservation after GKS remains a challenge, and long-term hearing preservation is still one of the major concerns of GKS treatments, particularly in younger patients.

Concerning surgery, advanced skull-base operative techniques with better intraoperative neurophysiological monitoring have facilitated gross total resection and functional preservation (facial and hearing) [3]. Considering that, for small VS, when managed by an experienced team, most patients will have a good facial function outcome regardless of the treatment choice (GKS, surgery), attention should be focused on the long term-hearing outcomes.

Hearing outcomes have been reported as more durable after surgery than after GKS [4,5] with variable hearing preservation rates. Some authors have reported good outcomes with speech discrimination over 50% [6,7]; others have observed poor long-term results with speech discrimination at 10 years under 3% [8]. In a recent systematic meta-analysis, hearing preservation was observed in 57% to 59% of the patients after follow-up at 47 months [9] and at least 5 years [2]. Yet, authors have reported significant variations in hearing preservation rates for patients with VS across series. In the most recent meta-analysis, the outcomes were analyzed irrespective of the device (linear accelerator, cyber knife, and gamma knife), and the specific population (intracanalicular VS with evidence of progression and good hearing) was not individualized.

In rare cases of incomplete central compensation, an active treatment may be indicated to control incapacitating vertigo. However, data on vestibular dysfunction after GKS are scarcely reported even in systematic meta-analyses.

In this study and in our practice, observation is our first choice of management with a clinical and audiological control at 6 months and an MRI control at 9 months. Without evidence of progression, follow-up is maintained with clinical and audiological control every 6 months and MRI control at 12 months. When there is evidence of clinical or radiological progression or the presence of incapacitating vertigo, we propose surgery via MFA or GKS. In our experience, functional outcomes after surgery are better with Koos 1 tumors than Koos 2 tumors, essentially for hearing preservation [10]. All patients are informed about the possible side effects of MFA and GKS, especially lack of tumor control or residual tumor, hearing loss, and facial nerve dysfunction. For these small tumors, facial nerve dysfunction risk is very low (<0.5%) with either surgery or GKS. Therefore, attention should be focused on the long-term evolution of hearing, as reported by Carlson et al. (follow-up of 9.3 years) [11].

The aim of the present study is to report the evolution of hearing after GKS for progressing intracanalicular VS with good hearing (speech discrimination > 70% and pure-tone average (PTA) < 30 dB HL). We also evaluated potential prognostic factors for hearing preservation after GKS.

## 2. Materials and Methods

A retrospective review of the outcomes of intracanalicular VS patients who underwent GKS between 2010 and 2020 was conducted at our institution. The inclusion criteria were as follows: (1) intracanalicular VS with evidence of progression either clinically or radiologically; (2) good hearing, defined as a pure-tone average (PTA) ≤ 30 dB HL and a speech discrimination score (SDS) ≥ 70% on the tumor side (AAO-HNS class A); and (3) a 3-year or longer follow-up (Table 1).

Audiometric data were recorded at the time of pre-GKS and the most recent follow-up appointment, including the PTA—calculated as an average of the hearing thresholds at 500 Hz, 1 kHz, 2 kHz, and 3 kHz—and the SDS (%)—evaluated using 50 monosyllabic words presented at a comfortable loudness level (speech intelligibility level of +35 dB) [12].

To report the hearing outcomes post-treatment, scattergrams were created using the PTA together with the SDS, as recommended by the Hearing Committee of the AAO-HNS for hearing reporting standards. Demographic data, hearing outcomes, and treatment measurements were extracted and analyzed. The GKS treatment metrics were measured using planning scans. The metrics included dosimetry, conformity of the treatment, cochlear dose, and the maximum cochlear dose. Since 2018, patients have been treated with the ICON gamma knife (the Gamma Knife 4C previously).

Pre-GKS MRI were performed on all the patients. Post-GKS MRI were performed every year for the first 3 years, every 2 years for the next 4 years, and then every 3 years. Absence of control of the GKS was defined if the volume increased after the first 3 years of follow-up.

Statistical analysis was performed using Jamovi version 2.3.26. The quantitative data were described using the range, mean, standard deviation, and median. The qualitative data were described using the number and percentage. The significance of the obtained results was judged at the 5% level. Statistical analysis included univariate analysis of variance (ANOVA).

## 3. Results

A total of 41 patients underwent GKS for VS with evidence of progression, initial good hearing (PTA ≤ 30 dB HL and SDS ≥ 70%), and at least a 3-year follow-up. Audiometric data were available for all of them. The average age of the patients undergoing GKS was 54 years (median: 55.0, standard deviation: 11.0, range: 30–74). Of the patient data set, 44% of the tumors were localized to the right side, and 56% to the left. The average volume of the tumors was 201 mm^3^. An extension of the tumor to the cochlear aperture at the lateral end of the internal auditory canal was noted in 49% of the cases.

All patients had planned treatment of 11.0 to 12.0 Gy at the 50% isodose line (mean dose: 11.6 Gy). The mean dose to the cochlea was 5.05 Gy (median: 4.90, standard deviation: 1.82, range: 1.60–8.80). The mean minimum cochlear dose was 3.41 Gy (median: 3.10, standard deviation: 1.32, range: 1.10–6.40). The mean maximum cochlear dose was 7.50 Gy (median: 7.80, standard deviation: 2.43, range: 2.20–11.8).

All the patients had good hearing as defined by an average PTA of 22 dB HL (median: 24, standard deviation: 7.01, range: 6–30) and an average SDS of 96% (median: 100, standard deviation: 6.73, range: 80–100). The average follow-up was 5.02 years (median: 5, standard deviation: 1.71, range: 3–9).

All the tumors were controlled by the GKS based on the last MRI follow-up; one patient after GKS still complained from incapacitating vertigo. No facial palsy was noted after GKS.

After a 5-year median follow-up, 34.1% of the patients maintained good hearing (AAO-HNS class A) on the side of the lesion (Table 2); 56.1% of the patients maintained serviceable hearing defined as post-GKS AAO-HNS class A and B.

Interval paired *t* test analysis showed a statistically significant (*p* < 0.01) decrease in the PTA and SDS. As shown in Table 3, hearing had a tendency to decline over time even if some patients were able to maintain class A hearing after a long follow-up.

**Figure 1:**
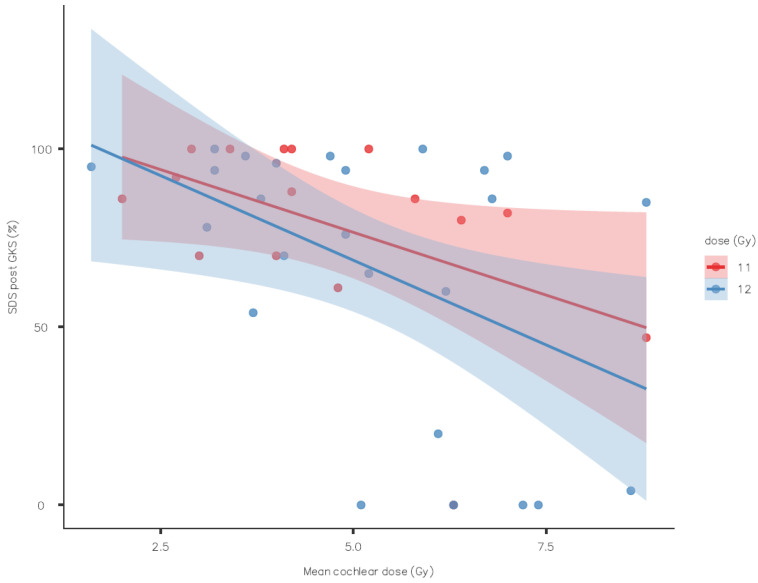
Speech discrimination score outcome by mean cochlear dose (standard error as shaded area).

**Figure 2:**
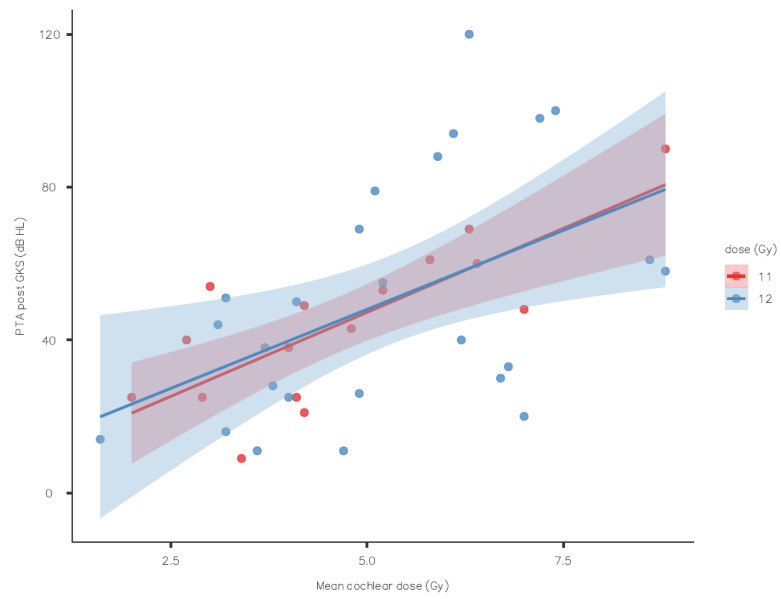
Pure-tone average outcome by mean cochlear dose (standard error as shaded area).

The evolution of the speech discrimination score showed no statically significant association with the mean dose at the 50% isodose line, but there was a tendency to have better hearing with 11 Gy (Figure 1) only for the SDS, not the PTA (Figure 2).

The mean cochlear dose was statistically associated with the final AAO-HNS class (*p* = 0.001): better hearing outcomes were associated with lower cochlear doses (Table 4). The same profile was noted with the minimum and maximum cochlear doses.

**Figure 3:**
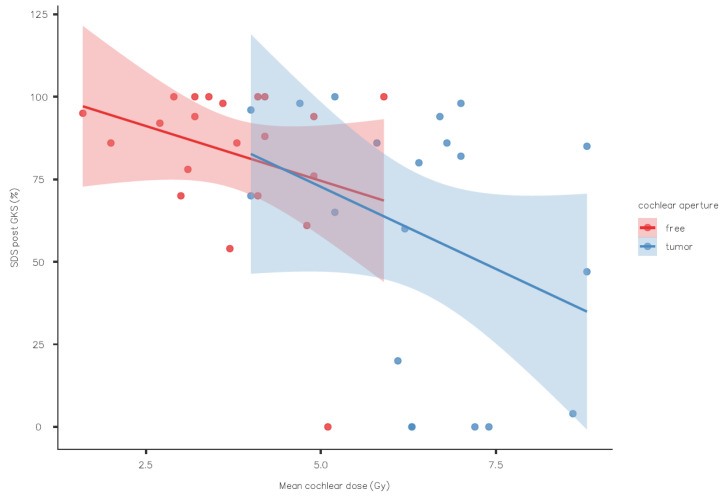
Speech discrimination score outcome by mean cochlear dose with or without tumor in the cochlear aperture (standard error as shaded area).

The mean cochlear dose was also statistically associated with the lateral extension of the VS (cochlear aperture free of a tumor or not). The mean cochlear dose was 3.73 Gy when the lateral end of the internal auditory canal was free of a tumor, 6.42 Gy when it was not (*p* < 0.001) (Figure 3). This cochlear dose difference certainly had an impact on the SDS post-GKS: the mean SDS was 82.9% when the lateral end was free versus 58.5% when it was not (*p* = 0.023).

## 4. Discussion

This single-center, retrospective study analyzed the hearing outcomes of GKS for patients presenting progressing VS with good hearing. After a median follow-up of 5 years, almost 35% remained in class A, and 56.1% kept serviceable hearing. Hearing deterioration has been reported to accelerate between years 5 and 10 [8]. Some teams argue to manage these tumors conservatively since hearing outcome is better with the “wait and scan” strategy [1] but others argue that the hearing continues to deteriorate regardless of tumor growth [3]. Concerning tumor growth, studies tend to demonstrate that up to 50% of the tumors show significant growth with time [13,14]. However, the patient will have to be treated if the tumor is growing, with higher risk of hearing deterioration when the tumor is larger.

Defining what is a success in terms of hearing preservation is subjective. The level of preservation considered satisfactory may be discussed, as well as defining the optimal follow-up duration. Niranjan et al. [7] with 42.5% of the patients remaining in class A after GKS with a median follow-up of 2 years and 4 months, concluded that GKS is a minimally invasive first-line management option for patients with intracanalicular tumors and provides high rates of hearing preservation with minimal morbidity. At the same time, hearing preservation outcomes after surgical removal in up to 75% of patients have been reported with longer follow-up [10,15,16]. Due to the reproducibility of the GKS technique, one may expect better homogeneity of the hearing outcomes after GKS treatment for a similar tumor volume with a similar treatment plan (mean dose at 50% isodose line, cochlear dose) when compared to surgical series, whereas the experience and technique may vary from one surgeon to another.

Concerning intracanalicular tumors and GKS, few articles have focused on treating patient with good hearing, and yet intracanalicular tumors with good hearing are diagnosed more and more frequently. Meta-analyses are not generally able to isolate this clinical situation. For example, Sughrue et al. reported that hearing preservation was 62% with small tumors (volume ≤ 1.5 cm^3^), but no details were given concerning the intracanalicular tumors with good initial hearing [17]. Comparing the evolution of hearing before and after GKS, Yomo et al. even argued that GKS may have a possible protective effect on hearing evolution [18]. They reported that a maximum cochlear dose of less than 4 Gy was the sole prognostic factor for hearing preservation. Additionally, GKS has been reported as able to improve hearing in 23.5% of a series of patients with intracanalicular VS [6] only in pure-tone audiometry without any report of speech intelligibility. In this study, tumor growth control was obtained in 91.2% of the cases, but the authors reported cases with facial nerve dysfunction (4/136 patients). A final serviceable hearing preservation rate was reported in 78.2% of the patients, whereas others reported a preserved serviceable hearing rate of 50% [19]. In that study, the authors explained the high level of hearing preservation by the dose reduction (up to 11.5 Gy) and other gamma plan strategies (angle selection, sector blocking, etc.) but gave few details on the methodology of hearing testing (levels of testing for speech intelligibility testing, type of word lists). Reporting hearing preservation only focusing on PTA may not be representative of the patient’s ability to understand speech [20]. Other teams with lots of experience in GKS have not emphasized the importance of these technical GKS parameters [5,7,8,21].

The reason for hearing deterioration after GKS in patients with VS has not been clearly documented. Many causes have been suspected: (1) direct radiotherapy damage to the cochlear nucleus, cochlear nerve, or inner ear (stria vascularis, sensory neuroepithelium, spiral ganglion cells); (2) vascular changes in the blood supply to the cochlea; (3) creation of adhesion between perineural tissues and tumors after GKS; and (4) transient volume changes in the intracanalicular tumor after GKS [22].

Historically, to increase hearing preservation, efforts have been made to reduce the radiotherapy dose [9]. Conversely, decreasing the tumor dose too much may lead to a lower tumor control rate. A systematic analysis reported a critical safe dose of 12.5 Gy at the 50% isodose line, but the cochlear dose may vary amongst authors [9,21]. Even with this level of tumor dose, the variability in the hearing outcomes (up to 25% in the hearing preservation score) is difficult to understand [6,7,21,23,24]. A low cochlear dose below 4 Gy has been recommended for optimal audiological outcomes [21,24]. However, it is interesting to note that hearing preservation seems to be better after GKS for facial nerve schwannomas [25] or for meningiomas extending into the internal auditory canal [26] with higher mean cochlear doses. However, in our series, the cochlear dose was the main statistically significant factor involved in the hearing outcomes. The patients remaining in class A had a mean cochlear dose of 4.01 Gy whilst the patients who moved to class D had a mean cochlear dose of 6.97 Gy.

The durability of hearing preservation should be reported as well because continued reduction over time in serviceable hearing after GKS is often reported [1,4,27], with an acceleration of the decrease between years 5 and 10 [8], whereas hearing preservation after microsurgery is thought to be more durable [5]. Moving from class A to class B is still beneficial for the patients because the patients with post-GKS serviceable hearing may benefit from a unilateral hearing aid on the tumor side, whereas CROS or BICROS are more indicated for patients with post-GKS classes C and D.

Hearing outcomes after observation have to be compared too. A multicenter retrospective review of 466 patients reported serviceable hearing at 94, 77, 66, and 44%, respectively, after 1, 3, 5, and 10 years of follow-up [28]. A similar study by Stangerup et al. reported 55% of serviceable hearing over a mean follow-up time of 4.7 years [29].

Due to the variability in hearing outcomes with GKS and surgical series, comparing these techniques is of utmost difficulty, and the optimal management strategy for small VS and good hearing remains controversial. Even if patients can make an informed decision according to their own priorities, clinician preferences may influence the treatment choice. Ideally, the same center should be able to propose surgical removal via MFA for intracanalicular tumors or GKS without any bias (difference of expertise in either treatment). Frequently, centers promoting GKS have less expertise in surgery via MFA (or another approach adapted for intracanalicular tumor) and “surgical” centers have less expertise in GKS. Thus, frequently, some centers suggest that one specific treatment is better than another treatment modality [3]. Even with the centers able to propose both options, publications may encompass selected cohorts of patients and bias (i.e., only operating on tumors with favorable characteristics for hearing preservation: absence of lateral extension, treating progressing tumors). This is the reason it is difficult to generalize any results obtained for a specific population.

## 5. Conclusions

In our experience, we propose both therapeutic options for progressing tumors even if ultimately it is up to the patient’s preference and priority. Surgery is our first option for patients who are younger with good physical status, good preoperative hearing status, prolonged incapacitating vertigo, and medial type VS because hearing preservation is better and more durable but with potential surgical morbidity. GKS is our first option for older patients or patients with poor physical status because of its low morbidity rate and good tumor control. Either way, it is necessary to have a discussion with the patient explaining the advantages and disadvantages of both techniques. Overall, in this selected population, GKS was able to preserve serviceable hearing in 56.1% of the patients with a tendency to decline over time. GKS in this series was associated with excellent tumor control and low facial nerve morbidity.

## Figures and Tables

**Table 1:** Representation of division of AAO-HNS hearing classification.

AAO-HNS Classification	PTA (dB HL)	SDS (%)
A	0–30	70–100
B	31–50	50–69
C	51+	50–69
D	any	0–49

**Table 2:** Hearing outcomes after GKS by AAO-HNS class.

AAO Post GKS	n	%
A	14	34.1%
B	9	22.0%
C	10	24.4%
D	8	19.5%

**Table 3:** Hearing outcome evolution at follow-up over time.

AAO-HNS	3 Years	4 Years	5 Years	6 Years	7 Years	8 Years	9 Years
A	2	4	1	4	1	1	1
B	4	1		1	2	1	
C	1	3	4	2			
D	2	3			2	1	

**Table 4:** Hearing results on the basis of mean cochlear dose.

AAO-HNS	n	Mean (Gy)	SD	SE
A	14	4.01	1.51	0.405
B	9	4.77	1.56	0.521
C	10	5.21	1.71	0.542
D	8	6.97	1.28	0.451

## Data Availability

The data presented in this study are available on request from the corresponding author.
